# RNAi‐Based Pesticides: Genetic Innovations for Sustainable Crop Protection and One Health

**DOI:** 10.1002/ggn2.202500063

**Published:** 2026-03-05

**Authors:** Jisheng Liu, Guy Smagghe

**Affiliations:** ^1^ School of Life Sciences Guangzhou University Guangzhou China; ^2^ Institute of Entomology Guizhou University Guiyang China; ^3^ Department of Biology Vrije Universiteit Brussel Brussels Belgium

**Keywords:** biopesticides, double‐stranded RNA (dsRNA), gene silencing, nanoparticle delivery, One Health, RNA interference (RNAi), sustainable agriculture

## Abstract

Conventional chemical pesticides have long protected global crop yields, but their environmental and health impacts, including contamination, biodiversity loss, residue accumulation, and pest resistance, demand sustainable alternatives. RNA interference (RNAi) represents a genetic breakthrough in crop protection, enabling precise, sequence‐specific silencing of essential pest genes via double‐stranded RNA (dsRNA). Innovations such as nanoparticle‐based delivery, host‐induced gene silencing (HIGS), and spray‐induced gene silencing (SIGS) have transformed RNAi into a practical, field‐ready technology. Integration with multi‐omics and computational biology accelerates target discovery and enhances specificity and scalability. Beyond pest management, RNAi applications extend to vector control and pollinator protection, exemplifying One Health principles. RNAi‐based pesticides therefore, redefine sustainable agriculture through molecular precision and cross‐sectoral genetic innovation.

## Graphical abstract

RNAi‐based pesticides offer precise, gene‐specific pest control using dsRNA to silence essential insect genes. Advanced delivery systems (nanoparticles, SIGS, HIGS) and multi‐omics‐driven target discovery enable effective, scalable, and environmentally safe crop protection, supporting biodiversity, human health, and One Health‐aligned sustainable agriculture.

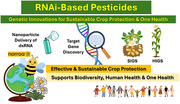

## 1 RNAi in Crop Protection: A Turning Point Based on Genetic Intervention

The introduction of chemical pesticides in the mid‐20th century revolutionized global agriculture by dramatically reducing losses caused by insect pests and other pathogens. However, decades of extensive pesticide application have revealed profound ecological and societal costs. The rapid evolution of resistant pest populations, coupled with the disruption of natural ecosystems and the persistence of toxic residues, has generated increasing concern regarding environmental and human health [1, 2]. In parallel, growing global food demand and climate‐driven shifts in pest distribution underscore the urgent need for innovative, sustainable, and precise pest management strategies.

Against this backdrop, genetic technologies such as RNA interference (RNAi) have emerged as transformative tools for crop protection. First described in *Caenorhabditis elegans* as a sequence‐specific gene silencing mechanism [3] and subsequently recognized as a conserved regulatory process across eukaryotic organisms [4], RNAi has been adapted to selectively target vital pest genes through the application of double‐stranded RNA (dsRNA) molecules [5, 6, 7]. Unlike broad‐spectrum chemical pesticides, RNAi offers exceptional specificity, reducing off‐target effects on beneficial insects and non‐target organisms while addressing the rising problem of resistance associated with conventional chemical interventions. Embedded within the broader One Health framework, RNAi‐based pesticides exemplify the integration of molecular genetics, plant science, and ecological stewardship, providing a platform that safeguards crop productivity, promotes biodiversity conservation, and supports human and environmental health simultaneously [7, 8, 9].

RNAi‐based technologies thus represent a paradigm shift from conventional chemical approaches toward molecularly precise interventions that harness genetic knowledge to meet the demands of sustainable agriculture. They provide not only a mechanistic solution to pest management but also a platform for cross‐sectoral innovation, encompassing agriculture, environmental protection, and public health [8, 9].

## 2 RNAi‐Based Pesticides: Molecular Mechanisms and Genetic Manipulations

The canonical RNAi pathway is well established, involving the recognition and processing of dsRNA by Dicer, loading of small interfering RNAs (siRNAs) into Argonaute proteins, and the formation of the RNA‐induced silencing complex (RISC), which directs sequence‐specific mRNA degradation [10, 11]. Beyond this foundational understanding, recent studies have revealed new dimensions of RNAi biology that can be leveraged for innovative pest control applications. High‐resolution structural analyses of Dicer‐R2D2 complexes have uncovered mechanistic details that enable the rational design of synthetic dsRNAs with enhanced stability, more efficient processing, and improved durability, thereby increasing the effectiveness of RNAi‐based interventions [12]. Comparative genomic studies have also revealed substantial variability in systemic RNAi pathways across insect lineages, highlighting opportunities to exploit natural differences for lineage‐specific pest control strategies and to enhance delivery efficiency [13, 14, 15].

In parallel, advances in synthetic biology have allowed the construction of customized RNAi vectors and programmable RNA scaffolds, where siRNA sequence design and structural optimization critically determine interference effectiveness, for multiplexed gene targeting, as well as integration with CRISPR‐based genome editing systems for enhanced precision and functional versatility [16, 17]. Such innovations have transformed RNAi from a mechanistic curiosity into a dynamic platform for genetic innovation, providing a highly adaptable toolbox for sustainable crop protection. Despite these advances, challenges remain in predicting RNAi efficiency across diverse pest species and environmental contexts, indicating a critical gap for future research. The integration of high‐throughput functional genomics and machine learning‐based predictive models may accelerate the identification of effective gene targets, enabling the rational design of RNAi agents tailored to specific pests and ecological settings.

## 3 RNAi Applications for One Health: From Crop Protection to Public Wellness

**FIGURE 1:**
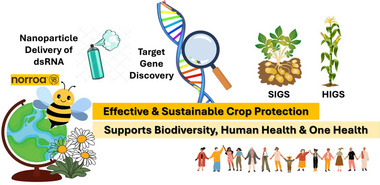
RNAi‐based innovation for sustainable crop protection and One Health. The essential target gene is silenced using dsRNA integrated with advanced delivery systems, such as nanoparticles, SIGS, and HIGS. Commercialized RNAi products, such as Norroa, demonstrate a safeguard for honeybee pollinators. Multi‐omics‐driven target discovery enables effective, scalable, and environmentally safe crop protection, supporting biodiversity, human health, and One Health‐aligned sustainable agriculture.

RNAi‐based technologies extend far beyond simple pest lethality, offering solutions that integrate genetic precision with ecological and human health objectives. In agricultural pest management, extensive research across multiple insect orders, namely from Lepidoptera, which exhibit relatively poor systemic responses, to highly RNAi‐responsive Coleoptera, has illuminated both the challenges and breakthroughs in achieving species‐specific targeting [18, 19, 20, 21, 22]. Notably, gene targets involved in cellular membrane trafficking consistently yield some of the most potent lethal effects observed in insect RNAi, underscoring their critical role in pest control efficacy [23]. Innovations in delivery systems, particularly the adoption of protective nanocarriers like chitosan and star polycation, have significantly enhanced the efficiency and stability of oral RNAi under field conditions (Figure 1) [24].

RNAi also holds promise for safeguarding beneficial insects. Targeted RNAi interventions have been developed to protect honeybee colonies from infestations by *Varroa destructor* mites, thereby reinforcing pollinator health and ecosystem resilience, both of which are essential for global food security [25]. Beyond pollinator conservation, RNAi has been deployed to mitigate the transmission of pathogens by insect vectors, demonstrating tangible public health benefits consistent with One Health principles (Figure 1) [26]. Expanding its reach, RNA‐based biopesticides are being adapted for aquaculture and animal health, targeting specific pathogens to reduce antibiotic usage and support responsible antimicrobial stewardship. Collectively, these applications illustrate RNAi's potential as a cross‐sectoral genetic technology that bridges agricultural productivity, ecosystem protection, and human health.

## 4 Beyond the Lab: Engineering RNAi Delivery for Durable Pest Resistance

Efficient dsRNA delivery remains a fundamental determinant of RNAi success. Traditional strategies such as host‐induced gene silencing (HIGS) involve transgenic crops expressing dsRNA targeting essential pest genes, conferring durable resistance against herbivorous insects (Figure 1) [5, 6]. Recent refinements, including mismatch‐corrected dsRNAs, have expanded the range and durability of HIGS by enabling multi‐target specificity and improved resistance management [27].

Complementing HIGS, spray‐induced gene silencing (SIGS) provides a flexible, non‐transgenic alternative that relies on exogenously applied dsRNAs (Figure 1). SIGS allows transient pest gene silencing through topical application and is increasingly compatible with regulatory frameworks due to its non‐transgenic nature. The integration of nanocarriers, such as chitosan nanoparticles, lipid‐based formulations, star polycations, and clay nanosheets, has markedly improved dsRNA stability, uptake efficiency, and environmental persistence [9, 28, 29, 30, 31, 32]. Alternative delivery strategies, including root irrigation, trunk injection, and hydroponic absorption, have proven effective for internal feeders and perennial crops, facilitating systemic RNAi throughout plant tissues [7, 33].

Emerging innovations are transforming RNAi delivery into a precision tool for field deployment. Smart formulations that combine dsRNA with conventional pesticides for synergistic effects, drone‐enabled precision spraying, and machine learning algorithms for optimizing dosage, timing, and environmental performance exemplify the next generation of delivery technologies. Future research will likely focus on environmentally responsive delivery systems, including pest‐activated dsRNA release and programmable nanocarriers, to further enhance specificity, reduce environmental impact, and ensure durability under field conditions.

## 5 Commercialization and Global Innovation Pathways

The transition of RNAi‐based pesticides from laboratory discovery to commercial application represents one of the most transformative developments in modern agricultural biotechnology. The commercial launch of SmartStax PRO corn, the first RNAi‐enabled crop targeting *Diabrotica virgifera virgifera*, provided proof‐of‐concept that RNAi can confer superior pest control while delaying resistance compared with Bt‐only hybrids [34, 35]. Parallel progress has been achieved with non‐transgenic sprayable dsRNA products, such as GreenLight Biosciences’ Calantha, which targets the Colorado potato beetle and exemplifies scalable RNAi deployment in conventional agriculture [18, 19].

Beyond crop protection, RNAi technologies have been extended to safeguard pollinators. For instance, the development of Norroa, a novel RNAi‐based miticide, demonstrates significant efficacy against *V. destructor* infestations in honeybee colonies (Figure 1) [25]. These commercial milestones demonstrate how foundational discoveries in RNAi biology have matured into globally relevant innovations that align with ecological safety, food security, and societal well‐being. Nonetheless, commercialization faces challenges in regulatory harmonization, public acceptance, and large‐scale production, highlighting the need for innovative business models and farmer support systems that facilitate adoption in diverse agricultural settings [8].

## 6 Challenges of RNAi‐Based Pesticides

Despite the remarkable promise of RNAi, several scientific, technological, and regulatory challenges remain. Interspecific variability in RNAi sensitivity continues to limit efficacy, as gene‐silencing efficiency can vary significantly among pest species [7, 22]. Furthermore, to counter resistance from target‐gene mutations, proactive resistance management strategies like gene rotation and multi‐target stacking are essential to ensure long‐term efficacy. Maintaining a balance between environmental safety and biological efficacy necessitates refined target screening, RNA structural modifications (such as incorporating chemical groups to shield dsRNA), and nanomaterial‐assisted delivery systems that enhance stability and uptake under field conditions [9].

Expanding RNAi applicability to a broader spectrum of crops, pest species, and plant pathogens, while integrating these strategies with conventional pest management, is essential for resilient and sustainable agricultural systems [36]. Crucially, widespread adoption hinges on demonstrating clear cost‐effectiveness compared to conventional pesticides and ensuring production scalability through streamlined, high‐yield manufacturing processes. Commercial success will also require market education, development of supportive supply chains, and robust outreach programs. Equally critical is the establishment of comprehensive regulatory frameworks and standardized assessment protocols to guide production, deployment, and post‐market evaluation. While early regulatory approvals set important precedents, the divergence of specific regulatory policies across different regions, such as the stringent environmental risk assessments required by the EU versus the product‐focused biopesticide frameworks in the US, underscores the need for global harmonization to ensure safety, ecological compatibility, and efficacy [37].

By systematically addressing these scientific, technical, and institutional challenges, RNAi‐based pesticides can mature into a sustainable cornerstone of modern pest management, with enhanced applicability, refined delivery systems, and regulatory alignment reinforcing their role in advancing One Health objectives [8].

## 7 RNAi and the Future of Genetics‐Informed One Health

RNAi‐based pesticides represent more than conventional crop protection agents; they constitute a transformative genetic platform that integrates genomics, bioinformatics, and biotechnology to create highly specific, environmentally sustainable, and adaptable solutions. Successful application across diverse sectors, including crop protection, pollinator conservation, and aquaculture, demonstrates the practical realization of One Health principles, simultaneously reducing chemical dependence, protecting biodiversity, and promoting ecosystem and human health (Figure 1).

Future innovation will depend on advanced formulations leveraging nanotechnology and synthetic biology to enhance field durability and precision, as well as artificial intelligence (AI) integrated pipelines, powered by machine learning and omics technologies, to accelerate the discovery of novel gene targets and optimize dsRNA design. For instance, AI‐driven models have been leveraged to predict RNA secondary structures and silencing efficiency, significantly streamlining the development of RNAi‐based agents [8], while also driving the implementation of AI in agriculture to optimize irrigation, refine the application of pesticides, and leverage AI for precise insect pest identification [38, 39]. Global regulatory harmonization will be critical to ensure safe, transparent, and equitable deployment, fostering public trust and responsible adoption. RNAi‐based pesticides exemplify how molecular genetics can redefine pest management, offering a cross‐sectoral, precision‐driven approach that advances sustainable agriculture, biodiversity conservation, and global health in an interconnected and rapidly evolving world.

## Conflicts of Interest

The authors declare no conflicts of interest.
